# Draft Genome Sequence of *Saccharomyces cerevisiae* NAM34-4C, a Lactic Acid-Assimilating Industrial Yeast Strain

**DOI:** 10.1128/genomeA.01145-13

**Published:** 2014-01-23

**Authors:** Takehiko Sahara, Kazuhiro E. Fujimori, Maiko Nezuo, Masatoshi Tsukahara, Yuki Tochigi, Satoru Ohgiya, Yue-Qin Tang, Kenji Kida, Hisataka Taguchi, Takashi Akamatsu, Yoichi Kamagata

**Affiliations:** a Bioproduction Research Institute (BPRI), National Institute of Advanced Industrial Science and Technology (AIST), Tsukuba, Japan; b BioJet Co., Ltd., Uruma, Japan; c Bioproduction Research Institute (BPRI), National Institute of Advanced Industrial Science and Technology (AIST), Toyohira, Sapporo, Japan; d Laboratory of Veterinary Physiology, Nippon Veterinary and Life Science University, Musashino, Tokyo, Japan; e Architecture and Environmental College, Sichuan University, Chengdu, China; f Graduate School of Science and Technology, Kumamoto University, Kumamoto, Japan; g Department of Applied Microbial Technology, Faculty of Biotechnology and Life Science, Sojo University, Kumamoto, Japan

## Abstract

We determined the genome sequence of industrial *Saccharomyces cerevisiae* strain NAM34-4C, which would be useful for bioethanol production. The approximately 11.5-Mb draft genome sequence of NAM34-4C will provide remarkable insights into metabolic engineering for effective production of bioethanol from biomass.

## GENOME ANNOUNCEMENT

*Saccharomyces cerevisiae* plays a central role in industrial ethanol production because of its ability to produce ethanol at high levels. *S. cerevisiae* NAM34-4C, a haploid strain derived from the industrial yeast strain KF-7 (1), possesses crucial traits required for industrial ethanol production from biomass, such as high productivity, thermotolerance, and acid tolerance. In addition, Wakamatsu et al. recently reported that *S. cerevisiae* NAM34-4C can produce a considerable amount of ethanol from d-lactate under acidic conditions (2, 3). There are no other reports regarding ethanol production from lactate by *S. cerevisiae*. Thus, *S. cerevisiae* NAM34-4C possesses a unique genetic background and useful genetic diversity for bioethanol production. To better understand the unique potential of *S. cerevisiae* NAM34-4C for bioethanol production, we determined its draft genome sequence by combining two next-generation sequencing technologies, the GS FLX Titanium system (Roche Diagnostics, Switzerland) and the SOLiD 3 system (Life Technologies, Inc., Carlsbad, CA). To fill the remaining gaps, Sanger sequencing of the amplicons was utilized.

The NAM34-4C genome was *de novo* sequenced with the GS FLX Titanium system to highly oversample the genome (31.2-fold coverage), with a total of 1,030,498 reads and the generation of a paired-end library, enabling the assembly of 539 contigs into 56 “supercontigs” (scaffolds) using the GS *De Novo* Assembler software (Roche). A genome of 11.5 Mb was covered by 56 scaffolds (*N*_50_ scaffold length, 409,700 bases). Whole-genome resequencing analysis of the NAM34-4C genome was performed using the SOLiD 3 system to improve the sequence quality of the draft genome, and 285,503,052 50-base reads were obtained. The SOLiD 3 reads were aligned to the scaffolds by BWA (4), Bowtie (5), and SAMtools (6) to detect the sequencing errors in the scaffolds obtained from GS FLX sequencing. As a result, 1,730 nucleotide differences were revised between the scaffolds generated by the GS FLX Titanium system and the reads generated by the SOLiD 3 system. We confirmed 389 of the intercontig gaps by PCR and closed them by Sanger sequencing of the amplicons with the 3730xl DNA analyzer (Life Technologies, Inc.).

Gene prediction and annotation were performed using AUGUSTUS software with the training set that is available for *S. cerevisiae* (7) and Exonerate software (8). The predicted proteins were searched against the curated open reading frames of the *Saccharomyces* Genome Database (SGD) (http://www.yeastgenome.org) (9) by BLASTp software (10), and matches were found for 5,696 proteins at an *E* value cutoff of 10^−6^.

### Nucleotide sequence accession numbers.

The nucleotide sequences of the *Saccharomyces cerevisiae* NAM34-4C draft genome have been deposited in DDBJ/EMBL/GenBank under the accession numbers BAUH01000001 to BAUH01000154. The version described in this paper is the first version.
